# Investigating Online Discussions About Cancer Screening on Twitter (Subsequently Rebranded as X): Corpus Analysis

**DOI:** 10.2196/90916

**Published:** 2026-08-28

**Authors:** Martin-Pieter Jansen, Hanneke Hendriks, Suzan Verberne, Gert-Jan de Bruijn, Enny Das

**Affiliations:** 1 Center for Language Studies, Radboud University Nijmegen, Nijmegen, Gelderland, The Netherlands; 2 Amsterdam School of Communication Research, University of Amsterdam, Nieuwe Achtergracht 166, Amsterdam, North Holland, 1018 WV, The Netherlands, 31 205250000; 3 Behavioural Science Institute, Radboud University Nijmegen, Nijmegen, Gelderland, The Netherlands; 4 Leiden Institute of Advanced Computer Science, Leiden University, Leiden, South Holland, The Netherlands; 5 Department of Communication Studies, University of Antwerp, Antwerp, Flanders, Belgium

**Keywords:** cancer, cancer screening, misinformation, social media, health communication

## Abstract

**Background:**

While cancer screening is proven to be effective in the early detection of the disease and early detection enables better treatment options, screening uptake has been declining. Research shows that online health information helps people to make health-related decisions. However, not all online health information is credible, and misinformation might play a role in people’s choice to take part in screening.

**Objective:**

This study aimed to analyze online discussions about cancer screening programs using corpus analysis. Specifically, we aimed to investigate the full dataset through corpus analysis and misinformation in a manually coded subset. This enabled us to study naturalistic discussions about cancer screening over time, what information people share, and how prevalent misinformation is in these discussions. We differentiated tweets on Twitter (subsequently rebranded as X) for cervical, breast, colorectal, and general screening.

**Methods:**

We extracted a corpus of 55,403 tweets from 2011 to 2023, tweeted by 22,493 users from a database containing over 5.9 billion tweets. We used specific search strings corresponding to different types of screening to gather our corpus. The corpus consisted of tweets, timestamps, hashtags, and shared URLs. We used a machine learning classifier trained on another dataset of tweets about cancer screening to automatically code whether a tweet fell within the scope of the study. We manually coded a randomly drawn stratified subset of 1200 tweets representative of the full corpus regarding year and screening program for the presence of misinformation.

**Results:**

Tweets were not uniformly distributed across different screening programs and over time (*χ*²_36_=4045.99, n=55,403*; P*<.001). Most tweets discussed population screening in general (n=35,199), and the volume of tweets increased around real-world events. Hashtags in the tweets predominantly focused on the screening programs that were discussed in those tweets. In our corpus, most shared URLs linked to other tweets (n=10,569) or news websites (n=2807). In our coded subset, information was shared in 679 tweets. Overall, 23 tweets contained misinformation. Topics in those tweets showed criticism toward the programs and policies, suspicions about conflicts of interest, and antivaccination beliefs regarding human papillomavirus (HPV). Most users used rhetorical questions, sarcasm, fearmongering, or expressed anger.

**Conclusions:**

Our findings reveal that cancer screening programs are actively debated across social media platforms. We observed that conversations tend to spike in response to real-world events, suggesting social media can serve as a valuable lens into public reactions to health policy changes. Link-sharing behavior was common, though we noted a tendency for sources to reference back to the same platform where discussions originated. Despite finding limited instances of misinformation, we caution that even modest amounts of inaccurate information may have meaningful consequences for public health messaging and screening uptake.

## Introduction

### Background

Cancer screening is a crucial aspect of cancer prevention and early detection. Detection provides better treatment options for cancer and early-stage cancer, thereby decreasing mortality rates [1]. The World Health Organization (WHO) recognizes three cancer screening programs as the most promising: breast, colorectal, and cervical programs [2]. Participation rates of at least 70% are recommended by the WHO for these programs to be effective [3]. However, an increasing number of people do not take part in screening programs, and some levels of participation are already below this threshold [4]. This makes it important to understand factors that may contribute to this nonparticipation. Earlier research already investigated determinants of this nonparticipation regarding demographics and psychological factors, but less is known about (user-generated) content on social media. Besides official sources, the internet has become one of the most prevalent sources of health information [5]. For instance, in the European Union, 85% of adults state that they use the internet for medical or health information [6].

On social media platforms, users share posts about their experiences, discuss opinions on health-related topics, or ask questions about procedures [7-9]. One of the issues with social media is that anyone can post anything, and that most information that is shared contains personal stories [10-12]. These personal stories are often not based on scientific evidence, but research shows that they are often presented as equally valid [10], and while social media has been found to increase access to health information and can function as a decision aid [13], concerns can be raised about the accuracy of information and the prevalence of misinformation in these discussions [14].

The fact that people frequently use social media to talk about health, and that such discussions are often based on personal narratives instead of scientific evidence, makes it vital to understand how cancer screening is talked about in online discussions and how misinformation may play a part in this, as this may influence people’s decision to take part in screening [15,16]. We investigate this by quantitatively analyzing a large corpus of 6 billion Dutch tweets on Twitter (subsequently rebranded as X). The study has 3 objectives. First, to gather insights into the volume of tweets and retweets about cancer screening. Second, to determine which types of resources are shared in the tweets. Research shows that this is one of the ways that people share news on Twitter (subsequently rebranded as X), and that the design of the platform makes it easy to do so [17,18]. Investigating which websites are shared can provide descriptive insights into the sharing of different types of external information on the platform. The third and final objective is to explore the amount of misinformation in tweets. By scrutinizing how prevalent misinformation is within cancer screening discussions in the Dutch Twitter (subsequently rebranded as X)-sphere, we provide insight into a part of health-related information that individuals might use to inform themselves about cancer screening programs.

### Online Health and Social Media

The internet has emerged as a dominant source of health information, with worldwide usage growing from 2.5 billion in 2013 to 5.2 billion in 2023 [19]. By 2024, an estimated 85% of European adults were using the internet to search for health or medical information [6]. Research indicates that multiple demographic groups in Europe show an increased likelihood of seeking health information online [20-22]. The European Union reported that in 2020, 76% of Dutch adults used the internet to look for health or medical information [23].

Not all online health information comes from medical or governmental websites. One of the types of online health information that people consume is discussions on social media [9,24,25]. Twitter (subsequently rebranded as X) is broadly used for the communication of health-related information, making it a very fitting platform in the context of the current work [26]. The platform had 3.1 million Dutch users in 2023. One of the main reasons that Twitter (subsequently rebranded as X) is an important platform to study is that it is embedded in everyday social and communicative interactions. Moreover, research using actual tweets allows us to study natural conversations in an unobtrusive manner [27]. Moreover, the platform’s role as a very public and real-time communication channel highlights the fact that it provides a window on contemporary society [28].

There are several actions that improve the spread or diffusion of messages (ie, tweets). Retweets may indicate the popularity of a message within a network and can be seen as a form of approval [29], especially when there is no comment that disagrees attached to the retweet. Another action that improves the spread of tweets on the platform is the use of hashtags. These word-labels help readers contextualize the message or provide emotional information that was not yet in the tweet [30,31]. Also, “hashtagged” words can result in promoted topics, or “trending topics” as they are called on the platform, gaining extra exposure and showing up in lists with trending words or topics [32].

Another way of sharing information on Twitter (subsequently rebranded as X) is via the attachment of a link to your tweet. This way of sharing news, or other information, means that one adds a URL to a tweet that leads to an external source. While research shows that more and more people rely on social media for their news consumption [33], Twitter (subsequently rebranded as X) often acts as a diffusing factor for news and information, where more traditional news is channeled differently compared to the presocial media age (eg, a tweet containing a link to an online news article) [34]. However, while sharing a URL to strengthen an argument might improve face validity to other users, the websites that are linked do not necessarily contain accurate information. This makes it important to investigate what types of websites the URLs in tweets lead to, as inaccurate information can misinform people, which can lead to lower intentions to take part in screening.

### Cancer and Social Media

Earlier work shows that online discussions about cancer screening contribute to people’s health-related decision-making, for example, whether or not to take part in screening [35]. However, it should be noted that differences between cancer screening programs exist in the role of internet information for a citizen’s decision to take part in screening. Specifically, research has shown that internet use has been positively associated with taking part in cervical cancer screening but that this was not the same for taking part in breast cancer screening [36].

While health information on the internet means that it may be more accessible for users, information sources that are shared on social media tend to be community-driven and grassroots-oriented (eg, a blog) and not news, from governmental sources, or from an established health institution [37]. Moreover, research shows that official health and news agencies, which typically provide reputable, scientific evidence-based information, are largely underrepresented in discussions on social media [38]. As a consequence, accurate information that would counter misinformation is less prevalent compared to false information. There are other concerns regarding online health information. Information can be one-sided or outdated, as has been reported in information about thyroid cancer [39]. But also, anyone with internet access, with or without expertise, can post information, which may open the door for potential misinformation. This raises concerns about the accuracy of online health information [14].

### Misinformation

A major issue associated with online health information is the growing spread of misinformation. Studies indicate that health-related misinformation has become increasingly common across digital platforms [40-42]. These worries are also underlined by the WHO, which speaks of an infodemic [43]. Research argues that features of social media, like the extensive use and relative ease of sharing information quickly, facilitate the spread of misinformation [44]. This is problematic, as studies show that users often encounter difficulties in recognizing misinformation [45,46]. Research in cancer-specific contexts reveals significant misinformation prevalence, with one analysis showing that 44% of highly engaged cancer-related social media posts contained false information [47]. Other work shows that tweets containing misinformation contain significantly more attractive language, fewer science-based facts, use more personal pronouns, and discuss trust issues and deception more than tweets containing correct information [48]. The occurrence of messages containing misinformation is especially pronounced regarding breast cancer screening on social platforms [16,49], where misleading content spans from exaggerated claims about foods’ anticancer properties to baseless fears about mammography risks. In this study, we do not assume that we know whether users share false information about cancer screening programs with the intention to harm others, which is often described as disinformation. Therefore, we use the definition of Wardle and Derakhshan [50] for misinformation as “when false information is shared, but no harm is meant.” Regarding “false information” in that definition, we follow Tan et al [51] and define this as: “what is considered to be correct or incorrect by expert consensus contemporaneous with the time period of this study.”

### Research Questions (RQs)

To systematically examine the nature of tweets and content about cancer screening in Twitter (subsequently rebranded as X) discussions, we pose the following research questions (RQs):

- RQ 1. What is the volume of tweets and retweets about cancer screening?
  - RQ 1.1. What are the differences in the volume of tweets and retweets, between types of cancer screening, and cancer screening in general?
  - RQ 1.2. What are the differences in the volume of tweets and retweets between types of cancer screening over time (per year/month)?
  - RQ 1.3. Which #hashtags are used most often, and does this differ between types of cancer screening, and over time?
- RQ 2. How is information from different types of resources (based on URLs) shared in the tweets?
  - RQ 2.1. Are there differences between cancer screening programs regarding what resources or types of resources are mentioned?
  - RQ 2.2. Is there a difference in the use of resources over time? And could these differences be linked to real-world events?
- RQ 3. What is the relative amount of misinformation regarding cancer screening programs in the tweets?
  - RQ 3.1. Are there differences regarding the amount of misinformation (1) over time, (2) between different cancer screening programs, or (3) per resource?

## Methods

### Study Design

This preregistered [52] content analysis study investigated how the Dutch cancer screening programs that are offered as population screening were discussed on Twitter (subsequently rebranded as X) between January 2011 and March 2023.

### Data Collection and Preprocessing

In this study, we used a corpus of tweets that we retrieved from a larger dataset that was developed in the TwiXL project [53]. This project started as the TwiNL collection and contains 50% of all Dutch-language tweets from January 2011 to March 2023. Please note that, while the TwiXL project is still aiming to retrieve tweets, this has become more complex and expensive after March 2023, when the platform decided to limit the API access for researchers [54]. At the time that we used the database to retrieve tweets, 5.9 billion tweets were accessible. We accessed the database via the TwiXL API in Python (version 3.11.1; Python Software Foundation) [55].

To ensure that we did not collect tweets that are outside of the scope of our project, we only downloaded tweets from the database based on four specific strings of keywords related to different types of cancer screening (ie, one for breast cancer screening, one for cervical cancer screening, one for colorectal cancer, and one for population-level cancer screening in general). For example, “mammogram” or “uitstrijkje” [swab] (Please see Multimedia Appendix 1 for the full search strings).

Our first step in data cleaning was deleting duplicate tweets based on the tweet ID. To ensure we only included Dutch-language tweets in the dataset, we used the langdetect Python library [56] to filter out non-Dutch content. Further manual investigation of the data revealed some noise in the langdetect output, caused by shortened URLs, hashtags, and usernames. Therefore, we decided to only exclude the tweets that were labeled as English, as manually assessing those tweets showed that the package was able to correctly label those tweets. Our final exported data consisted of 100,384 tweets. To ensure that tweets fell within the scope of this project, we used machine learning (a word-based Random Forest classifier) [57] to code whether tweets fell into the scope of the study (binary classification) [58]. The classifier was trained on a manually labeled dataset consisting of 1629 tweets.

We evaluated the classifier on a held-out test set of the labeled data. Precision at a threshold of .50 was .73 for inclusion, with a recall of .95. For exclusion, the precision was .67 with a recall of .24. As we wanted a higher precision (ie, less irrelevant tweets), we set the classification threshold for the ‘inclusion’ label at .70. At this threshold, the precision of the model was .81 for inclusion, with a recall of .77; for exclusion, the precision was .54 and the recall was .60. We focus more on precision than recall, as for this project we deem the correctness of positive predictions more important than the ability to find all relevant instances. Moreover, there are more inclusion tweets compared to exclusion tweets, meaning that precision is a more meaningful metric than accuracy. Our final corpus consisted of 55,403 tweets, tweeted by a total of 22,493 users.

We first investigated whether tweets were replies to other tweets by looking if a tweet started with an ‘@,’ we added a binary variable in our data indicating if this was true. We then did the same to see if tweets were retweets by looking if a tweet started with “RT.” Second, we used the rapidfuzz Python library [59] to calculate a similarity score (0‐1) based on the Levenshtein distance. Manual assessment of the tweets shows that retweets had a similarity ratio of >.85. We added a binary variable to indicate whether a tweet was a retweet if any of the conditions were true.

We cleaned the scraped URLs in the corpus. Twitter (subsequently rebranded as X) uses shortened URLs if a link is added to a tweet (eg, bit.ly). We used the requests Python library [60] to expand the URLs, and extracted the top-level domains via the tldextract Python library [61] for further analysis of the mentioned source domains.

### Coding Procedure

We manually coded a stratified random sample of 1200 tweets for the presence of misinformation (RQ 3). The sample was representative of the full corpus with respect to year and screening program. Tweets were double-coded by 4 coders making use of a codebook which is available on OSF in Dutch and as a version that is translated into English [52].

Before coding the full subset of 1200 tweets, the coders test-coded 125 tweets from these 1200. All tweets were double-coded in a rotational format, and all coders coded 50 tweets in total. This means that tweet 1‐50 were coded by coder 1, coder 2 coded tweets 25‐75, coder 3 coded tweets 51‐100, and finally coder 4 coded tweets 76‐100 and tweets 1‐24. The same rationale was applied to the coding of the other 550 tweets for each coder, resulting in all tweets being double coded.

First, the tweet was coded for inclusion (with three labels: inclusion, exclusion, and doubt). While we already used the specific search string and the machine learning classifier to make sure that tweets fell into the scope of the project, we still coded our subset for inclusion so that we were sure the correct tweets were included and excluded. Second, the tweets were coded for the cancer screening program (general, cervical cancer, breast cancer, and colorectal cancer) that was discussed in the tweet. General was coded if the tweet discussed population screening in general, or if more than one cancer screening program was discussed in the tweet. Third, tweets were coded for information sharing in general (yes-no). To be able to better distinguish misinformation in the tweets, it was coded whether information was shared in the tweet, regardless of whether this was correct information. Instructions clearly stated that this meant that information was presented as general, nonpersonal facts, with or without a source, and that it did not concern purely personal experiences. Finally, the tweets that were coded as having information shared in them were also coded for a suspicion of potential misinformation (yes-no). We did this because the coders could run into tweets that they were unsure of whether they contained misinformation (as our coders were no medical experts). The tweets that were coded for a suspicion of misinformation were exported and later manually assessed for the presence of misinformation by the main researcher (MJ) and other members of the project team and authors of this manuscript. To ensure we had as much data as possible, we included all tweets from the coded subsample that were coded for inclusion or doubt about inclusion at least once.

### Coding and Reliability

As indicators of agreement between the coders, we analyzed Cohen Kappa (κ) and the agreement percentage. For Cohen Kappa, we use the classification of Landis and Koch [62]. While we are reporting Cohen Kappa, we would like to underline that there are certain issues with the statistic. For example, in cases of class imbalance, a higher level of agreement can lead to lower test statistics, and the value can be inflated due to multiple answer options and/or coders [63,64]. This is why we choose to also report the plain percent agreement (PA) between our coders [65].

As described earlier, a briefing and test-coding meeting was organized by the main researcher before the coders started with test-coding of the first part of the subset of tweets. The tweets that were coded in the meeting were exported from the corpus and did not overlap with the tweets in the eventual subset of tweets. After this training, the coders started with the first subsample of 125 tweets. After this initial coding stage, the Kappa statistic and PA for the variables: Cancer Screening Program (κ=0.79, PA=85%), inclusion (κ=0.55, PA=77%), information sharing (κ=0.60, PA=75%), and suspicion of misinformation (κ=0.45, PA=68%) were calculated. Overall, the agreement according to the classifications of the Kappas was moderate to substantial, and the PA showed overlap in 76% of all cases.

After the coding of the first subset, another meeting was organized to discuss any disagreements, inconveniences, or things that were unclear that the coders came across while coding. After that, the other 550 tweets per coder were released. The final Kappas for the variables were fair, but far from desired: Cancer Screening Program (κ=0.95, PA=97%), inclusion (κ=0.45, PA=71%), information sharing (κ=0.38, PA=69%), and suspicion of misinformation (κ=0.36, PA=62%). After assessing the data, we calculated what would happen with the reliability indicators if each coder was left out of the analyses. We found that for one coder, the average Kappa increased by 0.1 if they were left out, which is what we did, and we finally ended up with the following reliability indicators: Cancer Screening Program (κ=0.95, PA=97%), Inclusion (κ=0.48, PA=71%), Information sharing (κ=0.51, PA=74%), and Suspicion of Misinformation (κ=0.61, PA=81%). These reliability indicators were deemed acceptable (and are moderate or better) and were much better than the indicators for all coders combined.

### Ethical Considerations

We received ethics approval from the Ethics Assessment Committee Humanities of Radboud University under approval number 2024‐1889 on June 27, 2024. While we did not collect usernames (these are pseudonymized), the combination of the following data in the dataset may lead to identification: tweet content, URLs (if shared in the tweet) (eg, hyperlinks to a news website), tweet ID, date of publication. If the tweet ID is used to find the tweet online, a user account could be identified. However, the usernames of the profiles that posted the tweets were pseudonymized and not anonymized. This also meant that if the tweet was a reply, retweet, or mention, the tweet included the username of the person that was mentioned, as it was part of the text of the tweet. It depends on what the user has made available whether this information can be used for the identification of an individual. The author ID or username will not be made publicly available. TwiXL does not include private tweets. Therefore, all information is publicly available. However, as the tweets in the database were scraped at different moments, it could be that tweets that are in the dataset have since been deleted by the user.

As we have collected tweets related to cancer screening, tweets that discuss the screening program itself were not expected to contain personal health information (ie, stating that they are invited to the screening, which is based on age groups mostly). However, the possibility existed that individuals did disclose personal health information. For instance, if a user tweeted that they received the results of their Pap smear and that these are positive and that further medical examination is needed. In that case, personal health information is collected.

Finally, the usernames of the profiles that posted the tweets were pseudonymized and not anonymized. This allowed us to see the structure of the conversations and how people interact on the platform and take part in (or leave) discussions. Moreover, if the tweet mentioned someone’s name or username in the text of the tweet (string), we also processed that information as it was part of the text of the tweet (eg, two friends who know each other and mention each other by their first name in a discussion and not their account names).

### Analysis

#### RQ 1: Volume of Tweets on Cancer Screening

For our first set of RQ’s, we counted the number of tweets and retweets per year to make crosstabulations to provide a descriptive overview. We did this for all tweets taken together, but also for each cancer screening program separately. We used chi-square tests to see if the number of tweets or retweets for each respective program and each year was higher or lower than the expected values and used a *P* value of *P*<.001 for these differences to be deemed significant. We did that because the sample size was very large and this may inflate the *P* value. We also calculated standardized residuals to examine which cells differ from the expected values in those cells. For the final part of the first RQ, we counted the number of hashtags in total, per cancer screening program and per year.

#### RQ 2: Shared Resources in Tweets on Cancer Screening

For the second RQ, we investigated information sharing in the tweets via the URLs that were added to tweets as attachments. We extracted the top-level domains as website names. Subsequently, we counted the times each website was included as an attachment and made rankings in total and per cancer screening program. Finally, we looked at the top 10 most used URLs over time.

#### RQ 3: Misinformation in Tweets on Cancer Screening

For the final RQ, we investigated the relative amount of misinformation in a stratified subset of 1200 tweets that were manually double-coded. We counted the relative amount of misinformation in the subset as well as the number of tweets potentially containing misinformation per cancer screening program.

## Results

### RQ 1: Volume of Tweets on Cancer Screening

**Figure 1.**
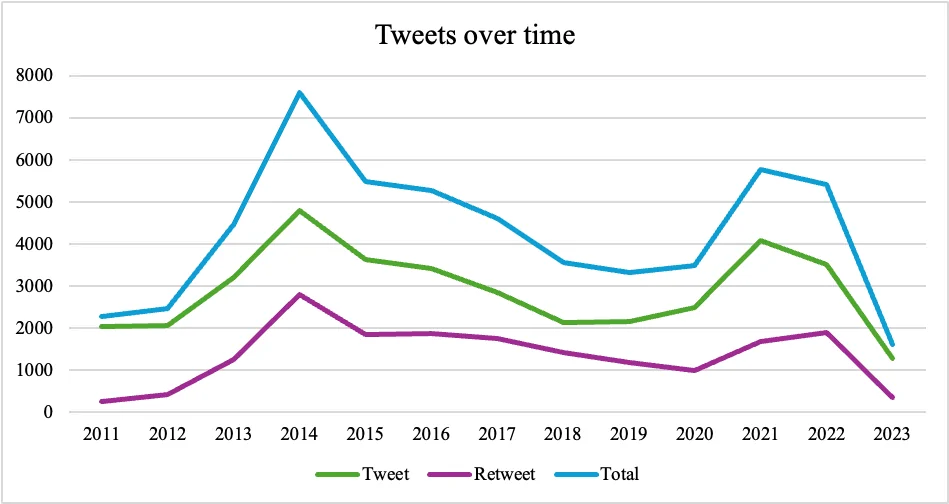
Volume of tweets, retweets, and regular tweets over time.

To address our first RQ, which asked: “What is the volume of tweets and retweets about cancer screening?,” this section examines the overall dataset. The dataset contained 55,403 tweets. In our sample, 10,312 out of 55,403 tweets (18.6%) started with a “@,” indicating that the tweet is a reply. We also investigated how many tweets were retweets in our dataset. In total, out of the 55,403 tweets in our dataset, 17,733 (32%) were retweets. To answer RQ 1, we investigated the differences in volume of tweets and retweets over time. Figure 1 shows the volume of tweets and retweets over time. Multimedia Appendix 2 shows an overview of this including standardized residuals.

Chi-squared tests showed a significant relation between year and whether a tweet is a retweet or not (*χ*²_22_=1720.88, n=55,403; *P*<.001). Examining the standardized residuals, we found that all cells besides the tweets in 2015 are overrepresented (SR>2) or underrepresented (SR<–2) in the volume of retweets from 2011 to 2023. The biggest deviations are the retweets in 2011, 2012, and 2023, where the volume was much lower than expected. Conversely, the number of tweets in 2011 and 2012, and retweets in 2018, were much higher than expected. We also found that the tweets were not distributed uniformly across the different cancer screening programs over time (*χ*²_36_=4045.99, n=55,403; *P*<.001).

**Figure 2.**
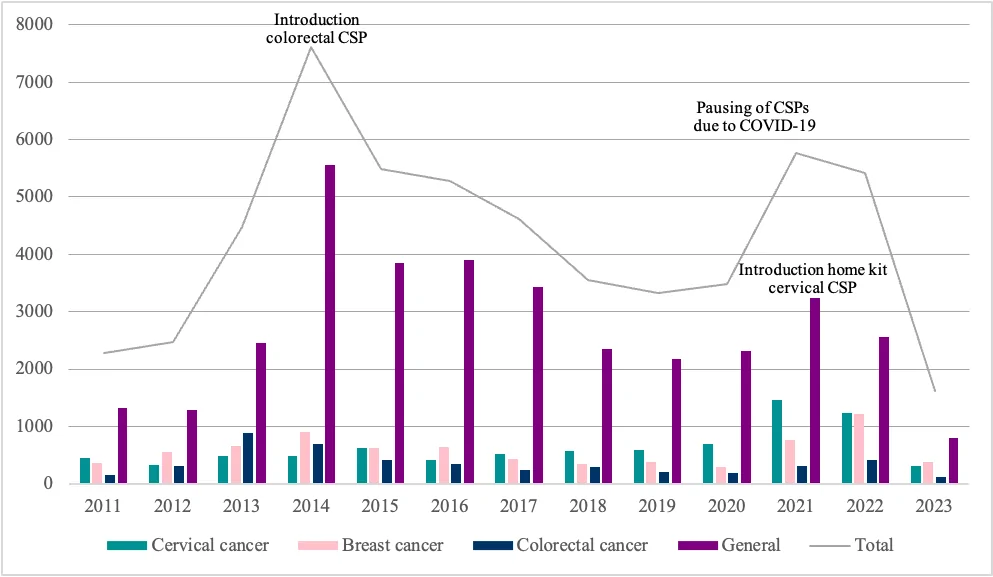
Number of tweets per cancer screening program from 2011‐2023; the line is total tweets about CSPs. CSP: cancer screening program.

Multimedia Appendix 3 shows the volume of tweets per cancer screening program over time, including standardized residuals. Standardized residuals show that the colorectal cancer screening program in 2013 was the biggest positive deviation from the expected number of tweets. This deviation co-occurred with the introduction of the population-based screening program for colorectal cancer, which started with a pilot in 2013 and was introduced a year later [66]. The second biggest deviation was the cervical cancer screening program in 2021; the third and fourth were the breast and cervical cancer screening programs in 2022. These increases in tweets co-occurred with the pausing of all population-based screening programs due to the allocation of health care resources to battle the COVID-19 pandemic [67], and the release of advice for the home kit for cervical cancer [68]. Figure 2 presents a visual overview of the tweets per cancer screening program per year, including those real-world events.

In total, 20,161 tweets (36.4%) of the 55,403 tweets in our dataset contained at least one hashtag. Most hashtags were used multiple times: 4685 hashtags were used a total of 68,003 times. A total of 20,161 tweets contained at least one hashtag; 8679 tweets contained at least two hashtags, and 3050 contained at least 3 hashtags. The top 5 most used hashtags in the dataset were (translated): breastcancer, populationscreening, cancerscreening, colorectalcancer, and breastcancerscreening. Table 1 shows the top 10 hashtags per cancer screening program (translated to English). The use of the top 10 hashtags overall, and how much they were used over time, can be found in Multimedia Appendix 4.

**Table 1.** Top 10 hashtags per cancer screening program.

	Cervical cancer		Breast cancer		Colorectal cancer		General	
	Hashtag	Frequency, n	Hashtag	Frequency, n	Hashtag	Frequency, n	Hashtag	Frequency, n
1	swab	291	mammography	377	coloscopy	218	populationscreening	2139
2	cervicalcancer	164	breastcancer	248	colorectalcancer	145	breastcancer	1589
3	populationscreening	71	Corona	174	populationscreening	53	Populationscreening	894
4	HPV	43	breastexam	92	stooltest	44	vacancy	892
5	coronavirus	41	mammogram	76	colonoscopy	37	colorectalcancer	815
6	health	37	health	56	health	36	news	689
7	hpv	37	populationscreening	44	knowledgesharing	36	cancerscreening	385
8	pauliencornelisse	35	news	42	colorectalexam	34	breastcancerscreening	370
9	GGD	31	thermography	39	onpc^a^	32	colorectalcancerscreening	304
10	NZa^b^	29	screening	30	complications	29	health	243

a onpc is a French talk show, manual assessment of these tweets showed that they were French, which could be because we only excluded English language tweets, please see Methods.

b NZa is the Dutch Healthcare Authority

### RQ 2: Shared Resources in Tweets on Cancer Screening

This section addresses our second RQ, which asked: “How is information from different types of resources (based on URLs) shared in the tweets?” We discuss the 10 most shared websites in more detail. In total, 23,579 (42.6%) tweets out of the 55,403 tweets in our dataset had a URL attached to them. The most shared website in the dataset was Twitter (subsequently rebranded as X) (n=10,569). If Twitter (subsequently rebranded as X) as a website is attached to a tweet, this means that people shared a link to another tweet attached to their original tweet. Please note that this is not the same as a reply or a retweet.

The second most shared website in the dataset is dpgmedia (n=1384). This means that news or an online article was shared in this tweet. DPG Media Group (“De Pers Groep” [The Press Group]) is one of the largest media publishers in the Netherlands. It owns some of the country’s largest national newspapers (eg, het Algemeen Dagblad, de Volkskrant, Trouw), and regional newspapers (eg, Brabants Dagblad, De Gelderlander, Tubantia). The main reason that DPG is the most shared website is that the publisher’s cookie pop-up is on that top-level domain (ie, dpg.nl), which leads to a website of the newspaper after agreeing to or rejecting the cookie pop-up.

**Figure 3.**
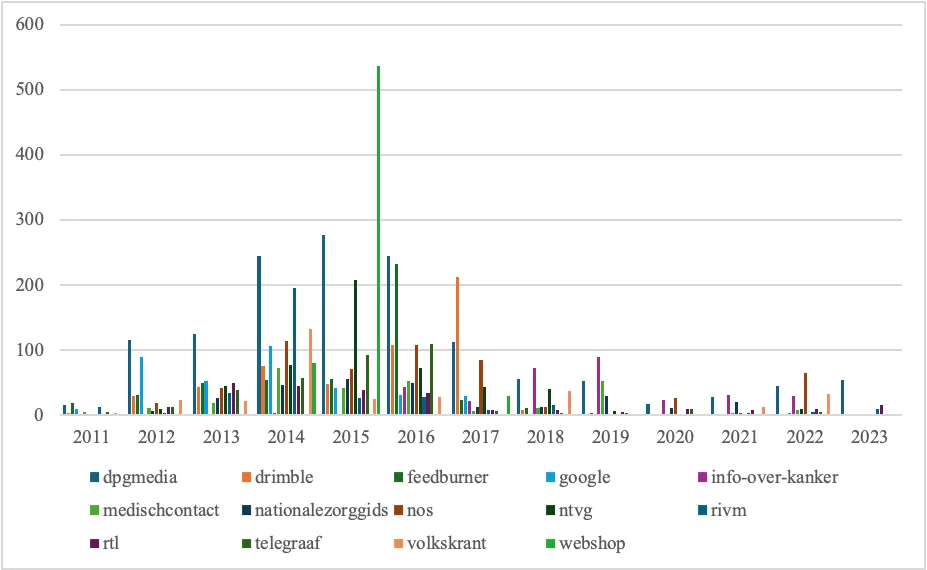
Shared sources over time.

The third most shared domain was “webshop” (n=650), which upon further inspection corresponded to affiliate redirect links from the marketing platform TradeTracker [69]. All tweets containing this domain were sharing job vacancies associated with the cancer screening programs, presumably distributed through a recruiting agency that used TradeTracker to track clicks before redirecting users to the actual vacancy page. Since the tweets in our dataset are pseudonymized; however, we cannot confirm this interpretation with certainty. As reflected in Figure 3, these tweets were concentrated almost exclusively in 2015, suggesting the agency discontinued use of this platform shortly thereafter. Because the URLs were no longer operational at the time of data collection, they resolved to TradeTracker’s general homepage. The fourth most shared website was NOS (n=547)*,* the Dutch public broadcaster, meaning that news or an online article was shared in the tweet. The fifth most shared website was Drimble (n=530). Drimble is a so-called news aggregation website. The website collects and shares news and information, with a special focus on local Dutch news.

The sixth most shared website was ntvg*,* the website of the Dutch Journal of Healthcare (Nederlands Tijdschrift voor Geneeskunde; n=504). The seventh most shared website was feedburner, a platform for managing RSS feeds (n=480). The eighth most shared URL was Google (n=366). The ninth most shared was Telegraaf, a Dutch newspaper (n=346). Finally, the tenth most shared website was RIVM, the website of the Dutch National Institute for Public Health and the Environment (n=343). Figure 2 shows the number of shares per website over time. In this figure, we excluded “twitter” (subsequently rebranded as X) as a shared resource, as the volume was so big that the other sources were not distinguishable anymore. We see one large peak for the Dutch Journal of Healthcare in 2015. Manual assessment of those tweets shows that there were tweets that were critical of colorectal cancer screening, and that those tweets shared a medical paper published in 2015 [70]. Other users were also critical of colorectal screening but shared a different article [71]. Finally, the journal posted an interview about the benefits and risks of colorectal cancer screening [72]. Those tweets resonated within the community and were retweeted relatively often. For more information, the crosstabs and figure including Twitter (subsequently rebranded as X) as a source, please see the appendix file on OSF [52].

In conclusion, our results show that most tweets that share a URL share one back to Twitter (subsequently rebranded as X). Besides that, 2807 tweets share links to news websites or sites belonging to a newspaper, and 504 tweets share a link to a scientific journal. Finally, the website of the body that organizes the cancer screening programs is the tenth most shared website but is still only shared 343 times in 23,579 tweets.

### RQ 3: Misinformation in Tweets on Cancer Screening

This section addresses our third RQ, which asked: “What is the relative amount of misinformation regarding cancer screening programs in the tweets?” The definition of misinformation we used can be found in the introduction. It should be noted that we do not equate criticism of cancer screening programs with misinformation. There is ongoing scientific debate about the effectiveness and harms of cancer screening, particularly for breast cancer, including substantive disagreement about overdiagnosis, cost-benefit ratios, and mortality benefits [73-75]. What we coded as misinformation were tweets making specific factual claims without any evidence base within the tweet itself, that is, claims not supported by a source, reference, or verifiable reasoning, and that contradict the scientific consensus at the time of the study.

To answer this RQ, we investigated a representative subsample of 1200 tweets (see Methods). The tweets were first coded for inclusion in the study, meaning that they contained references to the Dutch cancer screening programs. While we classified the tweets for inclusion in the project, we also manually coded the subset of 1200 tweets for inclusion and found that 924 tweets were discussing the Dutch cancer screening programs. In total, 679 tweets out of the 924 (73.5%) shared information about the cancer screening programs. From that subset, 266 tweets (39.2% of the subset) were flagged as potentially containing misinformation. Of this subset, 23 tweets (see OSF), which is 3.4% of the tweets in which information was shared, contained misinformation. Eight tweets pertained to cancer screening programs in general, and the other 8 tweets pertained to breast cancer screening. Four tweets pertained to the cervical screening program, and 3 tweets pertained to the colorectal cancer screening program. Due to the low number of tweets containing misinformation, other than preregistered, we do not differentiate beyond the different cancer screening programs or over time.

While a low number of tweets contained misinformation, we aimed to provide insight into the contents of those tweets. The tweets about cancer screening in general that contained misinformation included claims without an evidence base about the harms of population-level screening, unsupported assertions that the government tracks citizens who opt out of screening, and unsubstantiated suspicions about conflicts of interest (eg, pharmaceutical industry involvement). Tweets containing misinformation about breast cancer screening included claims without an evidence base in the tweet: assertions of ineffectiveness stated as established fact (eg, that screening does not save lives and is a colossal failure) without reference to the scientific literature, policy criticism framed as factual implication (eg, suggestively implying that pausing screening during COVID-19 caused large numbers of deaths), and medical confusion (eg, claiming that more mammograms directly cause more mastectomies and breast cancers). It should be noted that questions about the mortality benefit of mammography are genuinely contested in the scientific literature. We were therefore careful to code as misinformation only those tweets that made unqualified factual claims misrepresenting or distorting this debate, rather than engaging with it.

Tweets describing cervical screening with misinformation discussed unsupported distrust of HPV vaccination and unsubstantiated policy criticism. In this case, the policy criticism is misinformation that would be in favor of cancer screening rather than opposed to it: one user claimed that more frequent screening always equals fewer deaths, which oversimplifies the evidence. Tweets containing misinformation about colorectal screening made unsupported claims about harms caused by screening (eg, deaths due to screening attributed without evidence to the procedure) and expressed financial cynicism without factual grounding. Looking at the emotional and rhetorical styles in the tweets containing misinformation, most users used rhetorical questions, sarcasm, fearmongering, and expressions of anger. In sum, we did not find much misinformation in the subset of tweets. The misinformation that was present appeared to be primarily rooted in claims without an evidence base, nested within criticism and concerns about cancer screening, as well as unfounded assumptions about pharmaceutical industry involvement. Please note that these results may be anecdotal in the broader context of misinformation and cancer screening.

## Discussion

### Principal Findings

In this study, we investigated how the Dutch cancer screening programs were discussed on Twitter (subsequently rebranded as X) between 2011 and 2023. We did this by conducting a corpus analysis of an existing corpus of Dutch tweets. We had three overarching RQs. First, we looked at the volume of tweets about cancer screening. Second, we investigated how information from different sources is shared, based on attached URLs. Third, we analyzed a manually coded subset of tweets to scrutinize the relative amount of misinformation in the tweets.

Online discussions have the potential to contribute to people‘s thoughts about cancer screening programs, and these thoughts may predict their eventual choice whether to take part in screening. This study shows that cancer screening programs are discussed on social media, and that most tweets discuss cancer screening in general. Discussions increase around real-world events, underlining that social media discussions provide insights into events that happen in society. We find that people share news on social media by attaching links, but that these links also often circle back to the same platform. While we did not find much misinformation, we emphasize that these small amounts might still have large effects.

In the following, we discuss our 3 main findings. First, we found that of the total number of tweets, most tweets discussed cancer screening in general, and that this was true for all separate years as well. Further, we found that regarding the volume of tweets over time, tweets were not uniformly distributed across cancer screening programs and over time. More specifically, 2 peaks occurred. The first peak (2014, general and colorectal cancer) in the number of tweets in our data was in 2014, which co-occurred with the introduction of the colorectal cancer screening program. We see that compared to other years, the volume of tweets about colorectal cancer screening was higher in those years, as were tweets discussing cancer screening in general in 2014, which we treat as a combined, single peak. We tentatively conclude that the introduction of a new screening program (the colorectal program) also started discussions about screening in general.

The second peak in our data occurred in 2021 (2021, cervical and general cancer). This peak co-occurred with 2 real-world events. The first event was the pausing of the screening program in the Netherlands due to the COVID-19 pandemic in 2020. Research shows that only during the first peak of the COVID-19 pandemic, search volume for terms related to cancer screening programs on search engines decreased, and that the volume went back to the levels before the pandemic in a few months [76]. This might indicate that people only started discussing the pausing of cancer screening programs after a while, as they first heavily discussed the pandemic itself, as it was very newsworthy at that specific time and social media consumption increased [77]. The second real-world event that co-occurred around the second peak was the introduction of the home kit for the cervical program, which was introduced in 2021. In line with this, we saw an increase in the volume of tweets about cervical cancer screening, particularly in that year. This underlines that social media, besides traditional media, has an active role as a real-time means of information sharing [78], which has recently been confirmed in the case of cervical cancer on TikTok [79]. Moreover, it confirms earlier findings that social media and news media can complement each other regarding setting the public agenda [80], and other research showing that in regular (news) media, peaks in portrayals exist around real-world events in cancer screening [68], COVID-19 [77], and H1N1 [81]. This emphasizes that social media is an important channel to keep investigating, as it not only offers a window on society, but it is also both an important source and an important outlet for (health) information.

Our second main finding regarded information sharing in tweets by means of attaching links to other websites. We found that almost half of the tweets in our dataset included a URL. This way of sharing information on Twitter (subsequently rebranded as X) leads users to external sources, or other tweets on the platform, although it is not the same as retweeting or replying. For more than 40% of all tweets that had a URL attached, the link led to another tweet. In addition to referring to another tweet, around 12% of all tweets sharing a link contained a URL to a news website, or websites belonging to a newspaper. This brings us back to the previous argument that social media is not only an important source but also an outlet for news. In this case, the news that is published by publishers is shared by Twitter (subsequently rebranded as X) users, which may amplify the reach of news articles [17,18]. Further, in two percent of tweets accompanied by a URL, a link to an academic journal was shared, and in around one-and-a-half percent of tweets, a link to the governmental body that organizes the cancer screening programs was shared.

While we were unable to investigate the content of the linked tweets that were attached via the URLs, as they were not scraped in the original dataset, our results show that the attached links mostly led to reliable websites. This contrasts with other research showing that official health and news agencies were largely underrepresented in health-related discussions on Twitter (subsequently rebranded as X) [38]. However, we would like to emphasize that the percentages mentioned are relative to the tweets that referred to another website, which was around half of the original corpus, which means that the percentages would be half as large compared to the full corpus. This means that, looking at all tweets, most content is still user-generated and without an attached link to an external source, which confirms earlier research on cancer screening discussions on the platform [37].

Surprisingly, while there are rightful concerns about the sharing of incorrect health-related information on Twitter (subsequently rebranded as X) [40], we did not find websites with “alternative” or incorrect information in the attached URLs. However, as many URLs led back to tweets, they might be present indirectly, as the referred tweet may contain links to misinformation. In the scraped dataset, we could not access those tweets anymore, as they were not scraped in the original dataset. Moreover, many tweets do not exist anymore as people deleted older tweets or their accounts in general. This means that these findings should be considered in light of the dataset.

The third, and final, main finding of this work regards misinformation in tweets about cancer screening. Contrary to earlier research [47], which showed that 44% of the top 100 most popular cancer-related tweets contained misinformation, we found that in tweets where information was shared, less than 4% contained misinformation. Misinformation regarding cancer screening in general and the breast program was found eight times each, while four tweets about cervical screening and three tweets about colorectal screening contained misinformation. That we found more misinformation regarding breast cancer and general screening is partially in line with earlier work showing that breast cancer screening misinformation is most pronounced on social media [16], and Twitter (subsequently rebranded as X) specifically [49].

Before discussing the content of these tweets, it should be noted that criticism of cancer screening programs is not the same as misinformation. There is ongoing and substantive scientific debate about the effectiveness and harms of screening, particularly for breast cancer, including disagreement about overdiagnosis, mortality benefits, and cost-benefit ratios [73-75]. Policy criticism informed by this debate does not constitute misinformation as we define it. What we coded as misinformation were tweets making specific factual claims without an evidence base in the tweet itself, that is, claims not supported by a source, reference, or verifiable reasoning, and that contradict the scientific consensus at the time of the study.

The content of the tweets containing misinformation showed various subjects of discussion. Tweets with misinformation discussing population-based cancer screening in general mainly contained claims without an evidence base about the programs, their effectiveness, and the cost-benefit ratio of screening. Whereas tweets regarding the breast cancer screening program discussed the ineffectiveness of screening and policy failures without grounding, tweets regarding cervical and colorectal screening leaned more into existing conspiracy beliefs and showed beliefs of pharmaceutical industry involvement and financial cynicism (ie, the assumption that a few people get very rich over screening programs). This is in line with earlier research in general health information showing that different semantic features are found for different health topics [82]. However, we can only speculate about these ratios as we consider the volume of tweets containing misinformation in our subsample too small to draw conclusions. Moreover, as prior research found higher levels of misinformation in online health communication [40,41], we encourage future research to investigate misinformation across different domains of health communication and potentially compare these domains regarding the misinformation that appears. We also want to underline that several tweets that we classified as misinformation are indeed incorrect information but may be classified as personal narratives. These narratives are ungrounded, or based on memories of experiences (ie, mental models), or misperceptions that may not be in line with scientific consensus. They might not be tweeted with harmful intentions but may rather reflect a lack of knowledge [83,84].

As a final point, we want to emphasize that we do not mean to downplay the presence and risks of misinformation in online health communication. While we did not find many instances of misinformation in our data, we simultaneously do not want to underestimate the effects that even small amounts of misinformation can have. Research shows that cancer-related misinformation is designed to resonate through attractive language, personal pronouns, and deception [48] and that platforms facilitate the spread of misinformation through their respective designs [44]. These factors can strengthen one another, meaning that even small amounts of misinformation may have considerable effects.

### Limitations and Future Work

In this work, we made use of an existing corpus of tweets. While the dataset consisted of 50% of all Dutch-language tweets from 2011 to March 2023, we recognize that there is still a chance that we missed certain tweets by chance. Unfortunately, the dataset lacks metadata. This means that we could not investigate the popularity of tweets in the sample. It could be, for instance, that the tweets containing misinformation were retweeted more often or liked, leading to a bigger reach and larger diffusion effects compared to other tweets. Previous research showed that articles containing rumors are found to be shared over three times more often compared to scientific information [80]. Future research may investigate this through other studies that work with actual Twitter (subsequently rebranded as X) data. While we recognize that the company now wants enormous amounts of money for academic access, and that researchers are still unsure whether the datasets represent the whole platform, we encourage other platforms and projects to keep investigating Twitter (subsequently rebranded as X), as this is a great way to investigate natural conversations. Moreover, we encourage researchers to investigate other platforms in a similar manner, as cross-platform validations will strengthen our understanding of social media discussions. This is very needed in times of questionable transparency by platforms.

We were unable to distinguish between disinformation and misinformation because we did not know whether the users who wrote the tweets intended harm. It is possible that the inaccurate information shared stemmed from a lack of knowledge rather than a malicious intent. While both disinformation and misinformation can have significant effects, the distinction between them remains important, as deliberate choices can amplify harmful messages and expand their reach.

Regarding the authors of the tweets containing misinformation, it would be interesting to investigate who is sending these tweets. However, because the data were pseudonymized, we could not identify the types of accounts behind the tweets containing misinformation. As a result, we cannot say with certainty whether misinformation came from individual users, organized accounts, or alternative news platforms. Based on the writing style of the tweets, we suspect these were mostly individual users, but we cannot verify this. We encourage future research to investigate this further.

Further, we would like to encourage researchers to extend our results by investigating if they can be applied in different contexts. While at first it might seem obvious that our results could be applicable to other WEIRD (Western, educated, industrialized, rich, and democratic)–countries, they might even replicate in other geographical contexts. Moreover, it is also possible that other health communication-related subjects may replicate these results, and we encourage new studies combining the approach of working with existing data to investigate natural conversations about online health information.

Finally, results of this study do not predict how people make choices, nor are we able to make causal claims. While we did provide insights into factors that, supported by earlier work, might play a role in individuals’ health-related decision-making (ie, whether to take part in cancer screening programs), we were not able to establish causal claims and recognize that decision-making is a complex process. Future work may be able to investigate this process in a more causal way, or at least through retrospectively asking individuals what information they consider when making a choice to participate (or not), either qualitatively or quantitatively.

### Conclusions

Online discussions have the potential to contribute to people‘s thoughts about cancer screening programs, and these thoughts may predict their eventual choice whether to take part in screening. The current work presents an overview of discussions about cancer screening in the Dutch Twitter (subsequently rebranded as X)-sphere. This study shows that cancer screening programs are discussed on social media, and that most tweets discuss cancer screening in general. Discussions increase around real-world events, underlining that social media discussions provide insights into events that happen in society. We find that people share news on social media by attaching links, but that these links also often circle back to the same platform. While we did not find much misinformation, we emphasize that these small amounts might still have large effects.

## Supplementary material

10.2196/90916 Multimedia Appendix 1 Search strings in Dutch and English.

10.2196/90916 Multimedia Appendix 2 Tweets and retweets over time.

10.2196/90916 Multimedia Appendix 3 Tweets per cancer screening program over time.

10.2196/90916 Multimedia Appendix 4 Top 10 hashtags over time.

## Abbreviations

HPV: human papillomavirus
PA: percent agreement
RQ: research question
WEIRD: Western, educated, industrialized, rich, and democratic
WHO: World Health Organization
