# Supplementary material for: Investigating Online Discussions About Cancer Screening on Twitter (Subsequently Rebranded as X): Corpus Analysis
Source: JMIR Infodemiology. 2026 Aug 28;6:e90916. doi: 10.2196/90916 (PMC13536980; doi:10.2196/90916)
Supplement: Supplementary file 1 [file infodemiology-v6-e90916-s001.pdf]

Search strings in Dutch (original) and English (translated)

General cancer screening:

Dutch:

"kanker screening", "Kanker screening", "kankerscreening", "Kankerscreening", "bevolkingsonderzoek", "bevolkings onderzoek", "screeningsorganisatie", "vroegtijdige opsporing kanker", "vroegtijdige diagnose kanker", "vroegtijdige ontdekking kanker"

English:

"cancer screening", "Cancer screening", "cancerscreening", "Cancerscreening", "population screening", "Population screening", "screening organisation", "early detection cancer", "early diagnosis cancer", "early discovery cancer"

Colorectal cancer screening:

Dutch:

"ontlastingstest", "Ontlastingstest", "poeptest", "Poeptest", "colonoscopie", "Colonoscopie", "coloscopie", "Coloscopie"

English:

"stool test", "Stool test", "poop test", "Poop test", "colonoscopy", "Colonoscopy"

Breast cancer screening:

Dutch:

"mammografie", "Mammografie", "borstfoto", "Borstfoto", "mammogram", "Mammogram", "röntgenfoto borst", "Röntgenfoto borst", "borstonderzoek", "Borstonderzoek"

English:

"mammography", "Mammography", "breast photo", "Breast photo", "mammogram", "Mammogram", "X-ray breast", "x-ray breast", "breast examination", "Breast examination"

Cervical cancer screening:

Dutch:

"uitstrijkje", "Uitstrijkje", "uitstrijk", "Uitstrijk", "colposcopie", "Colposcopie", "baarmoederhalskankerscreening", "baarmoederhals kankerscreening", "Baarmoederhalskankerscreening", "Baarmoederhals kankerscreening", "baarmoederhals Kankerscreening"

English:

"smear test", "Smear test", "smear", "Smear", "colposcopy", "Colposcopy", "cervical cancer screening", "Cervical cancer screening"
