# Supplementary material for: Investigating Online Discussions About Cancer Screening on Twitter (Subsequently Rebranded as X): Corpus Analysis
Source: JMIR Infodemiology. 2026 Aug 28;6:e90916. doi: 10.2196/90916 (PMC13536980; doi:10.2196/90916)
Supplement: Supplementary file 2 [file infodemiology-v6-e90916-s002.pdf]

*Number of tweets and retweets over time (n), standardized residuals (SR) in italic*

| | Tweet | | Retweet | |
|---|---|---|---|---|
| Year | n | *SR* | n | *SR* |
| 2011 | 2032 | *12.28* | 246 | *-17.89* |
| 2012 | 2055 | *9.16* | 415 | *-13.36* |
| 2013 | 3217 | *3.13* | 1260 | *-4.57* |
| 2014 | 4809 | *-5.11* | 2805 | *7.45* |
| 2015 | 3632 | *-1.64* | 1857 | *2.39* |
| 2016 | 3416 | *-2.98* | 1871 | *4.35* |
| 2017 | 2845 | *-5.18* | 1766 | *7.55* |
| 2018 | 2144 | *-5.54* | 1410 | *8.08* |
| 2019 | 2151 | *-2.39* | 1180 | *3.49* |
| 2020 | 2500 | *2.59* | 991 | *-3.78* |
| 2021 | 4087 | *2.59* | 1685 | *-3.78* |
| 2022 | 3515 | *-2.76* | 1901 | *4.02* |
| 2023 | 1267 | *5.14* | 346 | *-7.49* |
| Total | 37 670 | | 17 733 | |
