# Supplementary material for: Investigating Online Discussions About Cancer Screening on Twitter (Subsequently Rebranded as X): Corpus Analysis
Source: JMIR Infodemiology. 2026 Aug 28;6:e90916. doi: 10.2196/90916 (PMC13536980; doi:10.2196/90916)
Supplement: Supplementary file 3 [file infodemiology-v6-e90916-s003.pdf]

Number of tweets per cancer screening program (CSP) over time (n), standardized residuals (SR) in italic

CSP	Cervical cancer		Breast cancer		Colorectal cancer		General cancer	
Year	n	*SR*	n	*SR*	n	*SR*	n	*SR*
2011	448	*6.15*	361	*2.93*	145	*-3.00*	1324	*-3.24*
2012	330	*-1.76*	551	*11.76*	312	*7.77*	1277	*-7.38*
2013	487	*-6.70*	662	*2.18*	882	*27.02*	2446	*-7.47*
2014	476	*-19.26*	897	*-4.27*	686	*2.58*	5555	*10.32*
2015	620	*-6.61*	615	*-4.78*	411	*-1.75*	3843	*6.02*
2016	416	*-12.98*	632	*-3.22*	338	*-4.51*	3901	*9.35*
2017	523	*-5.98*	431	*-7.81*	234	*-7.34*	3423	*9.12*
2018	570	*2.05*	347	*-6.18*	290	*-0.01*	2347	*1.87*
2019	584	*4.23*	384	*-3.22*	197	*-4.54*	2166	*1.08*
2020	690	*7.77*	299	*-8.05*	194	*-5.39*	2308	*1.91*
2021	1464	*21.08*	762	*-0.79*	305	*-7.66*	3241	*-7.04*
2022	1231	*15.36*	1215	*17.67*	407	*-1.67*	2563	*-14.97*
2023	316	*5.10*	370	*10.19*	122	*-0.84*	805	*-6.87*
Total	8155		7526		4523		35 199	
