# Supplementary material for: Investigating Online Discussions About Cancer Screening on Twitter (Subsequently Rebranded as X): Corpus Analysis
Source: JMIR Infodemiology. 2026 Aug 28;6:e90916. doi: 10.2196/90916 (PMC13536980; doi:10.2196/90916)

Table S1.

Top 10 hashtags for all cancer screening programs over time

Hashtag	2011	2012	2013	2014	2015	2016	2017	2018	2019	2020	2021	2022	2023
Populationalscreening	9	12	26	189	571	22	37	22	8	10	14	13	0
Cervical cancer	12	10	26	38	82	53	63	43	57	36	22	51	0
Populationscreening	41	61	178	396	227	357	429	203	136	136	193	155	0
Breastcancer	34	49	80	285	134	421	477	229	205	63	45	27	0
Breastcancerscreening	3	34	29	153	139	106	2	26	29	8	26	2	0
Colorectalcancer	25	25	205	470	216	65	101	71	21	42	98	54	0
Colorectalcancerscreening	1	3	39	209	77	80	31	8	20	1	3	0	0
Health	4	40	72	136	70	35	18	2	2	0	1	1	0
Cancer	14	48	30	52	87	24	25	6	7	10	13	2	0
Cancerscreening	0	1	28	56	31	46	42	191	100	65	43	61	0

Figure S1.

Top 10 hashtags for all cancer screening programs over time

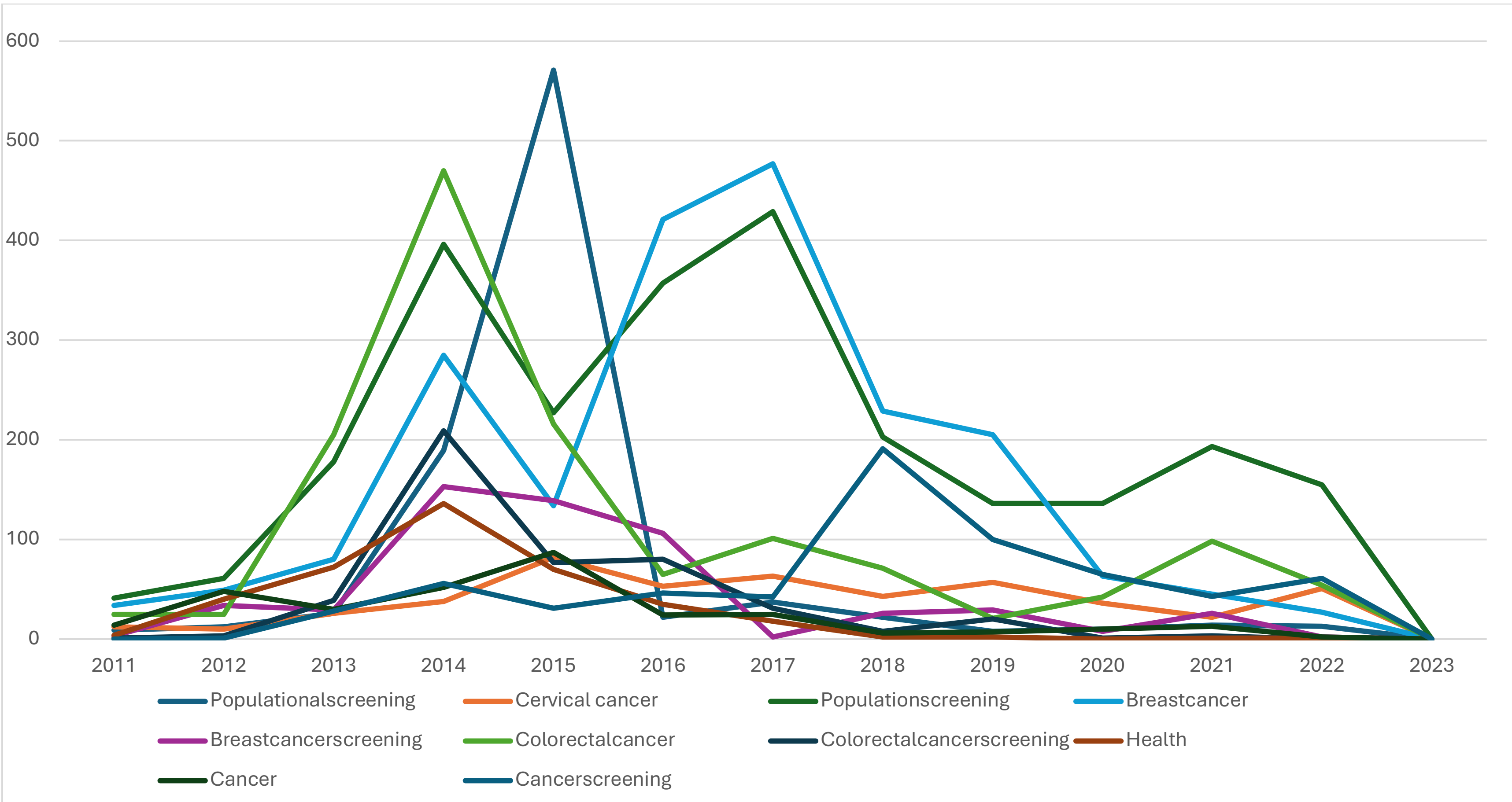

Supplement: Supplementary file 4 [file infodemiology-v6-e90916-s004.pdf]
